# Comparative Analysis of 198 SARS-CoV-2 Genomes from Iran and West Asia, February 2020 to December 2021

**DOI:** 10.30699/IJP.2023.557658.2935

**Published:** 2023-07-16

**Authors:** Saman Karamipour, Marzieh Mojbafan, Ramin Mazaheri Nezhad Fard

**Affiliations:** 1 Department of Genetics and Molecular Biology, Faculty of Medicine, Iran University of Medical Science, Tehran, Iran; 2 Department of Medical Genetics, Ali-Asghar Children's Hospital, Tehran, Iran; 3 Department of Pathobiology, School of Public Health, Tehran University of Medical Sciences, Tehran, Iran

**Keywords:** COVID‐19; Comparative genomics; Genome sequencing; Pandemic, SARS-CoV-2

## Abstract

**Background & Objective:**

Coronavirus disease 2019 (COVID‐19) is caused by severe acute respiratory syndrome coronavirus 2 (SARS-CoV-2), resulting in a worldwide pandemic. The first case of COVID‐19 was reported from Wuhan in the Hubei Province of China in December 2019; however, the disease's origin is still mysterious. Whole-genome sequence analysis is essential for monitoring the spread of infectious diseases as well as studying the pathogenesis and evolution of viruses. In this study, analysis of 198 fully sequenced genomes from Iran and West Asia was carried out to study mutations, phylogeny, amino acid changes, clades, and lineages of these genomes as well as comparison of these sequences with those of reference Wuhan genome of NC_045512.2.

**Methods:**

In total, 198 completely sequenced genome data from Iran and West Asia were collected from GenBank. Mutation detection was carried out using a trial version of CLC Genomics Workbench v.21.0 (QIAGEN, Germany). Online tools such as GISAID Mutations App and Pangolin were used for further analysis of the results.

**Results:**

In this study, several unique mutation sites were identified in the Iranian genomes (*n* = 8); positions 1397 G>A and 29742 G>T were the most frequent changes in more than 85% of the Iranian genomes. Mutation rate, mutation per sequence, and transition versus transversion for the Iranian genomes included 4.73, 14.14, and 1.6, respectively. Generally, C>T alteration was the most common substitution in all the sequences.

**Conclusion:**

The ORF1ab, N, and S were the genes with the most changes. The current data can help researchers predict future epidemics and establish better strategies to control viral pandemics.

## Introduction

In December 2019, a pneumonia outbreak with unknown causes emerged in Wuhan, Hubei Province, China. The outbreak rapidly spread worldwide. Primary genome sequencing showed that the causing agent was a novel coronavirus, which was later named 2019-nCoV or severe acute respiratory syndrome coronavirus 2 (SARS-CoV-2) by the World Health Organization (WHO) (1, 2, 3). The virus seemed to originate from bats with no clear transmission mechanisms to humans. The genome of 2019-nCoV included 89% nucleotide identity with bat SARS-like-CoVZXC21 and 82% with human SARS-CoV. However, studies showed that the two viruses were bound to similar host cell receptors of ACE-2. Furthermore, most of the characteristics of this novel coronavirus, such as its clinical and laboratory characteristics were similar to those of SARS; however, SARS-CoV-2 was sufficiently different from SARS-CoV, which could be considered a novel beta coronavirus. Bats are natural reservoirs of several zoonotic viruses, including henipaviruses, variants of rabies viruses, and coronaviruses (1, 4, 5, 6, 7, 8). From January 22, 2020, to October 11, 2022, more than 622 million confirmed cases of COVID-19, with nearly 6.56 million deaths, have been reported from 244 countries and territories (9).

Coronaviruses include a relatively large group belonging to the Coronaviridae family of the Nidovirales order. The Orthocoronavirinae subfamily is divided into four major alpha, beta, delta, and gamma coronaviruses genera. Coronaviruses usually cause infections in various domestic animals, from which alpha and beta coronaviruses predominantly infect mammals (10, 11, 12, 13, 14). Seven strains of coronaviruses can cause diseases in humans; however, these strains mostly cause mild infections. Other strains may cause life-threatening severe respiratory infections such as severe acute respiratory syndrome (SARS), Middle East respiratory syndrome (MERS), and severe acute respiratory syndrome coronavirus 2 (SARS-CoV-2) (14, 15, 28). Naturally, coronaviruses are enveloped and include non-segmented genomes. The virus particle includes crown-like glycoprotein spikes with 80–160 nm in diameter. Moreover, the viral genome includes pleomorphic positive-sense ssRNA molecules. This is the largest genome within the RNA viruses, ranging from 26–32 kb (10, 11, 13, 15). Familial clustering, demographic characteristics, exposure history, and other evidence have provided information on the virus's direct (person-to-person) transmission (10, 12). Compared to other severe coronavirus strains, such as SARS and MERS, that typically cause infections in humans, the mortality of COVID-19 is less (estimation of 4% for COVID-19 instead of 14–15% for SARS and 34% for MERS). However, the novel virus includes a higher transmission rate (morbidity), resulting in higher mortalities. The mortality rate is nearly 8% for people aged 70–79 years and 14.8% for those aged 80 years and older (8, 16, 17). The novel coronavirus genome shows 79 and 50% similarities with SARS and MERS viruses, respectively (8, 18).

Generally, SARS‐CoV‐2 is transmitted through respiratory droplets and feces. Unlike SARS and MERS, patients infected with COVID‐19 show high viral loads with no or mild symptoms (e.g., fever) (8, 19). Most of the viral genome includes ORF1ab at the 5’-terminal while the 3’-terminal consists of four structural proteins of a spike (S) that binds to the host receptors, envelope (E) that is essential for envelope formation, membrane (M) which infects cells, and nucleocapsid (N) (5'-ORF1a-ORF1b-S-ORF3-E-M-N-3'). The most common variant is detected in ORF1ab and ORF8 (2, 6, 9). The most vulnerable site for the entry and replication of SARS‐CoV‐2 is the respiratory tract (RT), especially the lungs. This possibly occurs due to the presence of angiotensin‐converting enzyme 2 (ACE2), a functional receptor for SARS‐CoV in human tissues. This receptor is further detected in the human brain, intestines, kidneys, liver, spleen, vessels, and skin; hence, the virus can affect multiple organs such as the circulatory system (CS), gastrointestinal tract (GIT), and RT. Symptoms of the infection appear within 5–6 days of incubation time and may result in death within 6–41 days (median of 14 days), depending on the patient's age and immune system condition (1, 20, 21, 22). The current study analyzed several published genomes of the virus to compare genome sequences of coronaviruses clinically isolated from Iranian patients with those isolated from West Asian patients. This analysis can help researchers improve further efficient molecular assays to decrease technical errors. Since single nucleotide polymorphism (SNP) in the viral genome can change its infection transmission rate and severity as well as the minimum age of the susceptible individuals, information from this study can improve personalized treatment policies.

## Material and Methods

**Acquisition of SARS-CoV-2 Genome Sequences**

A total of 350 complete sequences of SARS‐CoV‐2 from 11 countries were collected using the NCBI GenBank database (https://www.ncbi.nlm.nih.gov/labs/virus). Two major criteria were used for selecting sequences, including the removal of low-coverage (shorter than 29,000 bp) and low-quality (with more than 100 ambiguous nucleotides) sequences. Therefore, 198 SARS-CoV-2 sequences were finally used in the analysis. Since a small number of sequences were submitted by the countries, complete sequences were preferably selected. For further analysis of the selected sequences, reference sequences of an ancestral strain (NC_045512.2) from Wuhan, China, were used. The collection date was December 2019, the release date was July 2020, and the isolation source was severe acute respiratory syndrome coronavirus 2.

**Classification of the Genomes**

After grouping genomes based on their original countries, the CoVsurver Mutations App of GISAID (https://www.gisaid.org/epiflu-applications/covsurver-mutations-app) was used for the classification of genomes based on their clades. The reference sequence used by the website included hCoV-19/Wuhan/WIV04/19, which was identical to NC_045512.2. Furthermore, the Global Outbreak Lineages (Pangolin) COVID-19 Lineage Assigner (https://pangolin.cog-uk.io) was used to identify genome lineages.

**Phylogenetic Analysis of the Classified SARS-CoV-2 Genomes**

Classified genomes were used in phylogenetic analysis. First, CLC Software (trial CLC Genomics Workbench v.21.0, QIAGEN, Germany) was used to generate consensus sequences for each country. Then, a phylogenetic tree was plotted using previously reported methods and MEGA X Software (MEGA, USA). Briefly, MUSCLE was used for the alignment and maximum likelihood of the phylogenetic tree.

**Detection of Genomic Variances with Their Effects on Molecular Diagnosis**

Genomic variances were detected using CLC Software (trial CLC Genomics Workbench v.21.0, QIAGEN, Germany). This powerful software is developed to support a wide range of NGS bioinformatics uses. For example, CLC could combine quality control steps, adapter trimming, read mapping, variant detection, multiple filtering, and several annotations. Supported NGS platforms included Illumina, Ion Torrent, Oxford Nanopore, and PacBio.

**Dynamics of SARS-CoV-2 Mutations in Iran and West Asia**

Unique mutations in at least 30% of the Iranian genomes and common mutations in more than 80% of the cases were detected. These mutations were separately generated in the reference genome to analyze their effects on proteins. Moreover, major mutations in more than 70% of the West Asian genomes were detected and used for the analysis of their effects on protein sequences.

**Analysis of the Mutation Types**

Analysis of the mutation type for changes was carried out using CoVsurver (Protein Variation Effect Analyzer) (31), a helpful tool that predicts if an amino acid substitution or in/del affects the biological functions of a protein. Moreover, PROVEAN is useful for filtering sequence variants to identify nonsynonymous in/del variants predicted functionally important (https://gisaid.org/database-features/covsurver-mutations-app/).

## Results

**Classification of SARS-CoV-2 Genomes from Iran**

**Fig. 1:**
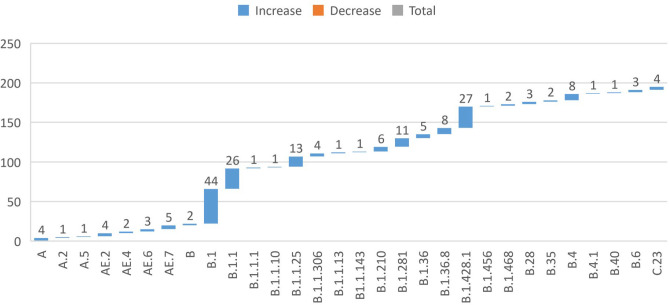
Distribution of the lineages in West Asia

From the first Iranian SARS-CoV-2 genome submission up to the time of this study, a total of 346 sequences were submitted in the GenBank. Eight out of 346 sequence sets were complete. One out of these eight sequences was excluded when the exclusion criteria were used. Of seven other sequences, six belonged to B.4 and one to B.1.210 lineages. Countries with B.4 lineage included the United Kingdom with 16.0%, Australia with 11.0%, Iran with 9.0%, India with 8.0%, and the United States with 8.0%. The B.4 lineage was called the Iranian lineage (https://cov-lineages.org/lineage.html?lineage=B.4). Overall, 30 lineages and subtypes were identified, of which, West Asian lineages of B.1 (22.0%) and B.1.1 (13.0%) were the most common lineages (Figure 1). The most common countries with B.1 lineage were the United States with 47.0%, the United Kingdom with 11.0%, Germany with 5.0%, France with 3.0%, and Canada with 3.0%. This lineage was a large European lineage, the origin of which corresponded to the Northern Italian outbreak in early 2020. Countries with the most common B.1.1 lineage cases were the United Kingdom at 36.0%, the United States at 12.0%, Japan at 7.0%, Germany at 5.0%, and Russia at 4.0%. This lineage was also called the European lineage.

**Fig. 2:**
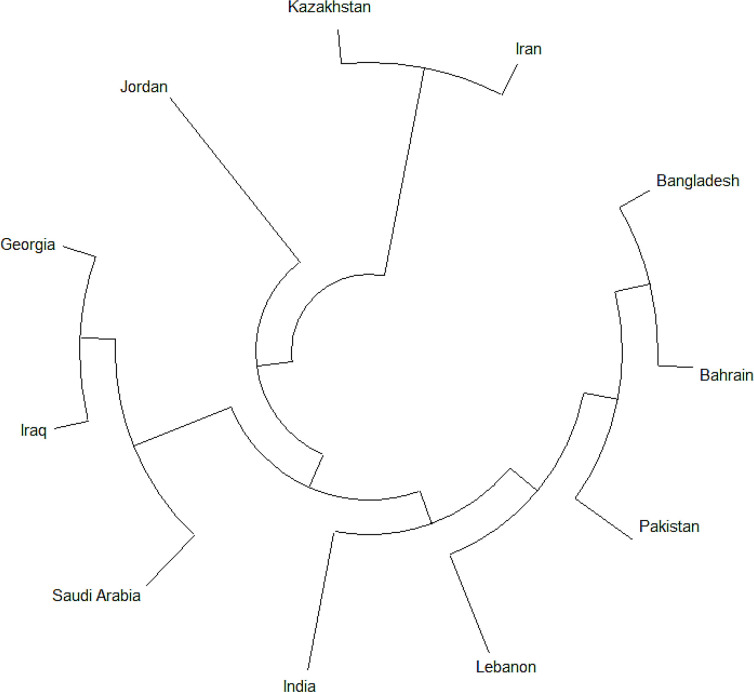
Phylogenetic tree of SARS-CoV-2 from West Asian countries after generating consensus sequences for each country

**Fig. 3:**
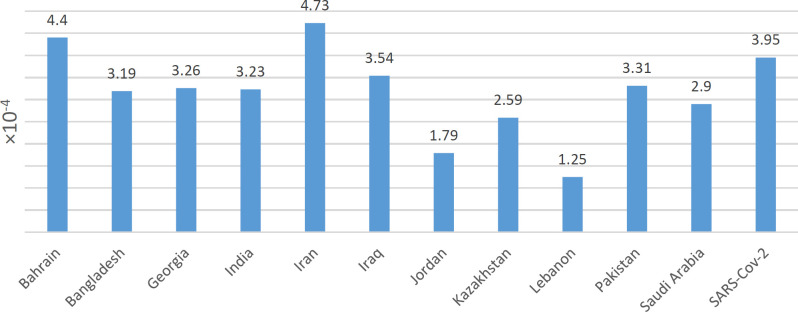
Distribution of the viral clades in various countries

Three Iranian sequences belonged to the L clades, while others belonged to others. The L clade referred to all genomes carrying reference alleles for all loci defined by G, GR, GH, S, and V clades. The O clade was a general group carrying sequences with no matches to the criteria (100). The Iranian genomes partially shared close ancestral relationships with Chinese SARS-CoV-2 isolates that began the COVID-19 pandemic. This was verified using a phylogenetic tree since the consensus sequence of Iran was located on a different arm of the phylogenetic tree compared to the consensus sequences of other countries (Figure 2) (101). Seven clades belonged to West Asia, including GH (46.5%), GR (31.5%), G (9.0%), O (4.0%), V (3.0%), S (3.0%), and L (3.0%) (Figure 3). The G clade was a variant of the D614G spike protein, significantly demonstrating a higher human host infectivity and better virus transmission efficiency. The GH and GR clades were the most common offspring of the G clade (102).

**SARS-CoV-2 Mutations Within the Genomes**

**Fig. 4:**
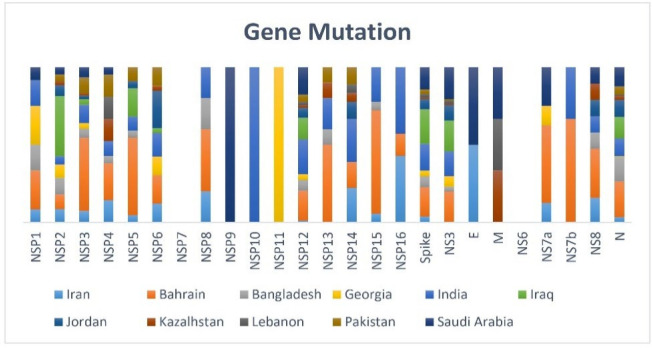
Gene mutation analysis for each country, showing viral gene mutations in each country.

Of the SARS-CoV-2 sequences submitted from Iran, 1397 G>A and 29742 G>T were the two most frequent mutations in more than 85% of the cases. Position 1397 was located in nsp2 and altered amino acid V198I, but Position 29742 was located in a non-coding region (3'UTR). Furthermore, 18083 G>T was another frequent change in 43% of the Iranian cases. Position 18083 was located in nsp6 and altered amino acid L37F. Interestingly, unique nucleotide changes were reported in the Iranian genomes compared to West Asian genomes. From these changes in the Iranian genomes, changes with approximately 30% frequencies were selected, including 3481 A>C, 8707 T>C, 18377 C>T, 20877 G>A, 21627 C>T, 22735 C>T, 28830 C>T and 29374 G>A. Of these altered positions, 3481 A>C, 8707 T>C, 22735 C>T, and 29374 G>A were synonymous and are located in nsp3, nsp4, S, and N genes, respectively (Figure 4). In addition, 18377 C>T was located in nsp14 (3’>5’ exonuclease), which resulted in T113I. The amino acid change (NSP14) of T113I occurred 1506 times (0.13% of all samples with NSP14 sequence) in 52 countries. The first strain with this amino acid change was isolated in February 2020 and named hCoV-19/Sichuan/SC-PHCC1-036/2020. England and the United States were the top countries with this mutation. Four types of mutation were observed in this position, from which the most frequent one was T113I (0.13%). The reference amino acid (T) included 99.87% frequency within all the sequenced genomes. The 20877 G>A was located in nsp16 (3’ > 5’ exonuclease) and altered amino acid G77R. The amino acid change of (NSP16) G77R occurred 177 times (0.01% of all samples with NSP16 sequence) in 14 countries. The first strain with this amino acid change was collected in February 2020 and included hCoV-19/France/PAC02003/2020. Denmark and the Netherlands were the countries with the highest frequency of this mutation. In this position, eight types of mutation were reported, from which the most spread was G77R (0.01). The reference amino acid (G) included 99.98% frequency within all the sequenced genomes. Moreover, 21627 C>T was located in S and altered T22I amino acid. The amino acid change (S) of T22I occurred 1624 times (0.14% of the samples with spike sequence) in 53 countries. The first strain with this amino acid change was isolated in February 2020 as hCoV-19/Germany/HB-RKI-I-014899/2020. England and Scotland were countries with the most spread rate of this mutation. In this position, nine types of mutations were reported, from which the widespread mutations were T22I (0.14%) and T22N (0.02%). The reference amino acid (T) included 99.84% frequency within all the sequenced genomes.

**Fig. 5:**
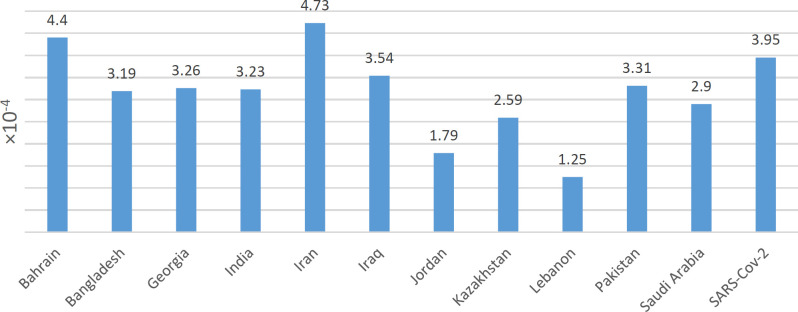
Mutation rates for each country. Iran with the highest mutation rate is compared to the other countries, calculated by average changes in each sequence per reference complete sequence length.

The 28830 C>T was located in N and altered amino acid S186F. Amino acid change (N) of S186F occurred 90 times (0.01% of all samples with N sequence) in 21 countries. The first strain with this amino acid change was isolated in March 2020 and named hCoV-19/Germany/NW-UBI-52/2020. The Republic of Ireland and the United States were reported as the countries with this mutation. In the highlighted position, nine mutations were reported, including S186Y (0.07%) and S186F (0.01%). The reference amino acid (S) included 99.91% frequency within all the sequenced genomes. Analysis of the West Asian genomes showed numerous changes. Of these changes, common changes in more than 80% of the cases were selected, including 241 C>T, 3037 C>T, 14408 C>T, 23403 A>G, and 25563 G>T. The 241 C>T mutation was located in a non-coding region (5'UTR), and 3037 C>T in NSP3 as synonymous mutations. Another 14408 C>T mutation was located in NSP12 (RNA polymerase) and altered P323L amino acid. The Amino acid change (NSP12) of P323L occurred 1144799 times (95.41% of the samples with NSP12 sequence) in 172 countries. The first strain with this amino acid change was hCoV-19/Italy/LAZ-TIGEM-6927/2021. England and the United States were the countries with the most prevalence rate of the mutation. Twelve types of mutations were detected in this locus, including P323L (95.41%), P323F (0.26%), and P323H (0.6%). The reference amino acid (P) was 4.22% frequency within all the sequenced genomes. Another mutation, 23403 A>G, was located in S and altered the D614H amino acid. The amino acid change (S) of D614G occurred 1159771 times (96.66% of the samples with S sequence) in 171 countries. The first strain with the highlighted amino acid change included hCoV-19/Colombia/ANT-CWOHC-VG-SEC00083E/2021. England and the United States were reported as top countries with the mutation. Nine mutations were investigated in this locus; the two highest occurred mutations were D614G (96.66%) and D614N (0.01%). The reference amino acid (D) frequency included 3.32% within all the sequenced genomes. The 25563 G>T mutation was located in ORF3a and altered amino acid Q57H. The amino acid change of NS3 Q57H occurred 270250 times (22.52% of all the samples with NS3 sequence) in 157 countries. The first strain with this amino acid change, isolated in January 2020, was hCoV-19/USA/AZ-TG808431/2020. The United States and England were countries with the highest rates of this type of mutation. In this position, 13 types of mutations were reported, with Q57H (22.52%) and Q57Y (0.02%) as the most frequent mutations. The reference amino acid (Q) included 77.45% frequency within all the sequenced genomes.

**Point Mutation Calculation in SARS-CoV-2 Sequences**

**Fig. 6:**
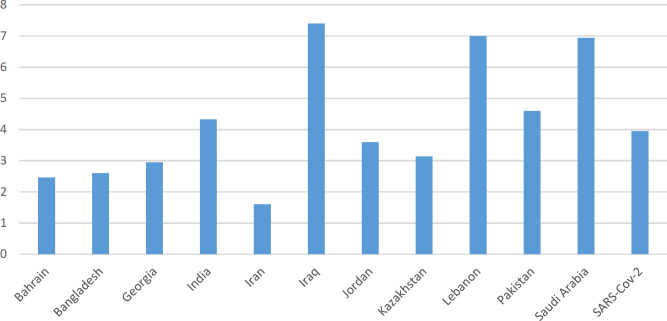
The ratio of transition to transversion for each country

The current study showed 73 mutation loci in Iranian genomes, of which 62 loci belonged to nonsynonymous mutations and 41 ORF1ab, nine S, and seven N based on isolate sequences from West Asia (Table 1). The transition/transversion rate was 1.6, and the G>A was the most spread substitution in all countries. Averagely, 14.14 point mutations were reported in each SARSCoV-2. The reference included 29,903 nucleotides; thus, the mutation rate was 4.72 × 10^-4^ per nucleotide (Figure 5). Genomes from West Asia showed 85 mutation sites. The transition/transversion was 3.96, and the C>T was the most substitution type (Table 2, Figure 6).

**Fig. 7:**
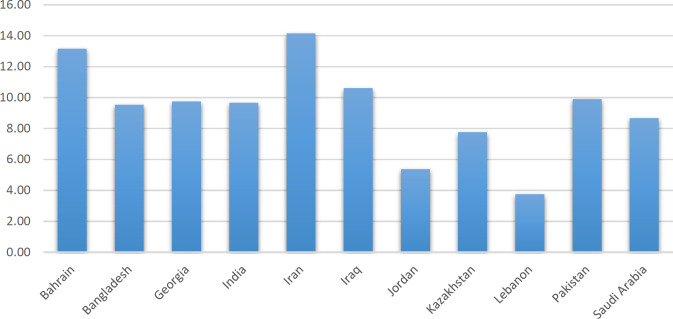
Changes per sequence for West Asian SARS-CoV-2 genomes

On average, 10.06 point mutations were detected in each SARSCoV-2 sequence set (Figure 7). The reference included 29,903 nucleotide sites; therefore, the mutation rate was 3.36 × 10^-4^ per nucleotide. Mutation rates and transition/transversion (Ts/Tv) for SARS-CoV-2 were 3.95 × 10^-4^ and 3.95, respectively (24).

**Table 1:** Distribution of mutations in West Asian isolates of SARS-CoV-2

Gene	Synonymous	Start	End	Length	No. of changes	Most change
5' UTR	-	1	265	264	-	-
leader protein	NSP1	266	805	539	13	-
NSP2	-	806	2719	1913	140	V198I, T85I
NSP3	-	2720	8554	5834	146	A994D
NSP4	-	8555	10054	1499	25	-
3C-like/proteinase	NSP5	10055	10972	917	23	-
NSP6	-	10973	11842	869	38	L37F
NSP7	-	11843	12091	248	0	-
NSP8	-	12092	12685	593	6	-
NSP9	-	12686	13024	338	1	-
NSP10	-	13025	13441	416	1	-
RNA polymerase	NSP11/12	13442	16236	2794	192	P323L
helicase	NSP13	16237	18039	1802	10	-
3' > 5' exonuclease	NSP14	18040	19620	1580	22	T113I
endoRNAse	NSP15	19621	20658	1037	19	-
2'-O-ribose methylteransferase	NSP16	20659	21552	893	10	-
S	Spike	21563	25384	3821	252	D614G
ORF3a	NS3	25393	26220	827	131	Q57H
E	envelope protein	26245	26472	227	3	-
M	membrane glycoprotein	26523	27191	668	3	-
ORF6a	NS6a	27202	27387	185	0	-
ORF7a	NS7a	27394	27759	365	9	-
ORF7b	NS7b	27756	27887	131	3	-
ORF8	NS8	27894	28259	365	22	L84S
N	nucleocapsid phosphoprotein	28274	29533	1259	216	S194L,R203K,G204R
ORF10	-	29558	29674	116	0	-
3'UTR	-	29675	29903	228	0	-
ORF1ab	-	266	21555	21289	636	-

**Table 2:** Maximum composite likelihood estimation of the nucleotide substitution patterns (%) in SARS-CoV-2 using 191 viral sequences

	A	G	T	C	In/del
A	-	17.1	1.1	0.21	
G	2	-	14.14	0.32	
T	0.86	0.65	-	2.7	4.7
C	2	0.054	53.56	-	

A, adenine; G, guanine; T, thymine; C, cytosine; in/del, insert/deletion

## Discussion

The current analysis of seven samples from Iran and 191 samples from West Asia showed that the Iranian genomes belonged to the B.4 lineage of L and O clades, while B.1 and B.1.1 were the most common lineages in West Asia. These findings showed that the Iranian samples' origin differed from West Asian countries. The phylogenetic tree verified the hypothesis that the Iranian genomes were different from those of other West Asian countries.

Based on these findings, it seemed that the origin of COVID-19 in Iran was directly linked to China (23). This study's whole-genome sequence analysis revealed that genomes isolated from eleven West Asian countries belonged to GH, GR, O, S, G, V, and L. This indicated the diversity of the infection in this region. In fact, GH and GR were the most widely affected ancestors of genomes within the West Asia genomes, while genomes of the V clade were rare. These results were verified by a study on the Asian SARS-CoV-2 clade in 2020 (24). Naturally, SARS-CoV-2 continuously changes its genome content, changing its characteristics and degrees of infectivity. Therefore, mutations can strengthen virus severity and infectivity. Of nonsynonymous unique changes in Iranian genomes, 21627 C>T of T22I substitution in spike protein was neutral with no effects on protein structures; however, 18377 C>T, 20887 G>A, and 28830 C>T, respectively, leading to T113I in NSP14 protein, G77R in NSP16 protein and S186F in N (nucleocapsid phosphoprotein), were deleterious and changed the protein structures. It has been shown that the NSP14 protein of coronaviruses has two major roles, including 1) replication fidelity in DNA viruses and proofreading that prevents lethal mutagenesis and 2) methyltransferase that contributes to mRNA capping. Technically, this protein is complex with its activator of NSP10. Similar to NSP14, NSP16 needs to be complex with NSP10 as a cofactor for proper activity, including critical functions for RNA cap formation. These functions affect the viral RNA stability. Naturally, N is a multifunctional protein that includes several roles in viral assembly, transcription, and forming complexes with genomic RNA (22, 25, 26). This evidence was possibly the reason why the Iranian genomes included the highest mutation rates compared with other West Asian countries.

Moreover, it has been shown that P323L mutations in regions of NSP12 and D614G from the spike protein are mostly mutated in the entire dataset and are globally dominant. Although mutations alter the structural stability of proteins by altering free energy, they are not harmful. This shows that mutations are not harmful but greatly affect the proteins' biological functions (24). Based on the literature, the G614 type variant includes a higher transfer capacity than the D614 type variant, associated with low RT-PCR cycle thresholds with no increases in disease severity. The P323L mutation resides in RNA-dependent RNA polymerase (RdRp) and alters intramolecular interactions of the protein as its stability changes (27). Results of this study have verified this information since D614G in spike, and P323L in NSP12 were the most frequent mutations in West Asian isolates. Of the most commonly nonsynonymous mutations, 25563 G>T that led to Q57H in ORF3a and 28881 G204del that led to in/del in N (nucleocapsid phosphoprotein) were deleterious. Analysis showed that Iranian isolates included more mutation rates compared to the other West Asian isolates. However, the mean rate of mutations from the West Asian isolates was close to the mean rate of the isolates from the rest of the world.

## Conclusion

The current study included an analysis of genomics variations and phylogeny of the SARS-CoV-2 sequences in Iran and West Asia, as well as their comparisons. Results demonstrated that most of the variations belonged to ORF1ab, N, and S that were linked to the virus replication and transcription, virion assembly, and host cell entrance. Furthermore, phylogenetic analysis showed that almost all Iranian genomes belonged to the B.4 lineage of L and O clades, while B.1 and G were the most common lineages and clades of the West Asian sequences. Data from this study in addition to those from other studies, can provide an opportunity to predict the virus behaviors and help medical communities control the pandemic.

## Funding

 None.

## Conflict of Interest

The authors declare no conflict of interest.
